# Benchmarking for biomedical natural language processing tasks with a domain specific ALBERT

**DOI:** 10.1186/s12859-022-04688-w

**Published:** 2022-04-21

**Authors:** Usman Naseem, Adam G. Dunn, Matloob Khushi, Jinman Kim

**Affiliations:** 1 School of Computer Science, The University of Sydney, Sydney, Australia; 2 Biomedical Informatics and Digital Health and Faculty of Medicine and Health, School of Medical Sciences, The University of Sydney, Sydney, Australia; 3 School of EAST, University of Suffolk, Ipswich, UK

**Keywords:** Bioinformatics, Biomedical text mining, BioNLP, Domain-specific language model

## Abstract

**Background:**

The abundance of biomedical text data coupled with advances in natural language processing (NLP) is resulting in novel biomedical NLP (BioNLP) applications. These NLP applications, or tasks, are reliant on the availability of domain-specific language models (LMs) that are trained on a massive amount of data. Most of the existing domain-specific LMs adopted bidirectional encoder representations from transformers (BERT) architecture which has limitations, and their generalizability is unproven as there is an absence of baseline results among common BioNLP tasks.

**Results:**

We present 8 variants of BioALBERT, a domain-specific adaptation of a lite bidirectional encoder representations from transformers (ALBERT), trained on biomedical (PubMed and PubMed Central) and clinical (MIMIC-III) corpora and fine-tuned for 6 different tasks across 20 benchmark datasets. Experiments show that a large variant of BioALBERT trained on PubMed outperforms the state-of-the-art on named-entity recognition (+ 11.09% BLURB score improvement), relation extraction (+ 0.80% BLURB score), sentence similarity (+ 1.05% BLURB score), document classification (+ 0.62% F1-score), and question answering (+ 2.83% BLURB score). It represents a new state-of-the-art in 5 out of 6 benchmark BioNLP tasks.

**Conclusions:**

The large variant of BioALBERT trained on PubMed achieved a higher BLURB score than previous state-of-the-art models on 5 of the 6 benchmark BioNLP tasks. Depending on the task, 5 different variants of BioALBERT outperformed previous state-of-the-art models on 17 of the 20 benchmark datasets, showing that our model is robust and generalizable in the common BioNLP tasks. We have made BioALBERT freely available which will help the BioNLP community avoid computational cost of training and establish a new set of baselines for future efforts across a broad range of BioNLP tasks.

## Background

The increasing amount of published biomedical literature, such as health literacy [1] and clinical reports [2] demands more precise and generalized biomedical natural language processing (BioNLP) tools for information extraction. Recent advances in natural language processing (NLP) have accelerated the development of pre-trained language models (LMs) that can be used for a wide variety of tasks in the BioNLP domains [3].

However, directly fine-tuning of the state-of-the-art (SOTA) LMs for bioNLP tasks, like Embeddings from Language Models (ELMo) [4], Bidirectional Encoder Representations from Transformers (BERT) [5] and A Lite Bidirectional Encoder Representations from Transformers (ALBERT) [6], yielded poor performances because these LMs were trained on general domain corpus (e.g., Wikipedia, Bookcorpus, etc.), and were not designed for the requirements of biomedical documents that comprise of different word distribution, and having complex relationship [7]. To overcome this limitation, BioNLP researchers have trained LMs on biomedical and clinical corpus and proved its effectiveness on various downstream tasks in BioNLP tasks [8–15].

Jin et al. [9] trained biomedical ELMo (BioELMo) with PubMed abstracts and found features extracted by BioELMo contained entity-type and relational information relevant to the biomedical corpus. Beltagy et al. [11] trained BERT on scientific texts and published the trained model as Scientific BERT (SciBERT). Similarly, Si et al. [10] used task-specific models and enhanced traditional non-contextual and contextual word embedding methods for biomedical named-entity-recognition by training BERT on clinical notes corpora. Peng et al. [12] presented a BLUE (Biomedical Language Understanding Evaluation) benchmark by designing 5 tasks with 10 datasets for analysing natural biomedical LMs. They also showed that BERT trained on PubMed abstracts and clinical notes outperformed other LMs which were trained on general corpora. The most popular biomedical pre-trained LMs is BioBERT (BERT for Biomedical Text Mining) [13] which was trained on PubMed and PubMed Central (PMC) corpus and fine-tuned on 3 BioNLP tasks including Relation Extraction (RE), named-entity-recognition (NER), and Question Answering (QA). Gu et al. [14] developed PubMedBERT by training from scratch on PubMed articles and showed performance gained over models trained on general corpora. They developed a domain-specific vocabulary from PubMed articles and demonstrated a boost in performance on the domain-specific task. Another biomedical pre-trained LM is KeBioLM [15] which leveraged knowledge from the UMLS (Unified Medical Language System) bases. KeBioLM was applied to 2 BioNLP tasks. Table 1 summarises the training corpora used in previous pre-trained biomedical LMs, whereas Table 2 presents a number of datasets previously used to evaluate pre-trained LMs on various BioNLP tasks. In our preliminary work, we showed that a customised domain-specific LM outperforms SOTA LMs in NER tasks [16].

**Table 1:** Data used in prior state-of-the-art studies compared to ours (BioALBERT)

Training corpus	BioBERT [13]	SciBERT [11]	BLUE [12]	PubMedBERT [14]	KeBioLM [15]	BioALBERT
General	$\checkmark$	$\times$	$\checkmark$	$\times$	$\times$	$\checkmark$
PMC	$\checkmark$	$\times$	$\times$	$\checkmark$	$\checkmark$	$\checkmark$
PubMed	$\checkmark$	$\times$	$\checkmark$	$\checkmark$	$\checkmark$	$\checkmark$
Clinical notes	$\times$	$\times$	$\checkmark$	$\times$	$\times$	$\checkmark$

**Table 2:** Comparison of the biomedical datasets in prior studies and ours (BioALBERT)

Datasets	BioBERT [13]	SciBERT[11]	BLUE [12]	PubMedBERT [14]	KeBioLM [15]	BioALBERT
Share/Clefe [17]	$\times$	$\times$	$\checkmark$	$\times$	$\times$	$\checkmark$
BC5CDR (disease) [18]	$\checkmark$	$\checkmark$	$\checkmark$	$\checkmark$	$\checkmark$	$\checkmark$
BC5CDR (chemical) [18]	$\checkmark$	$\checkmark$	$\checkmark$	$\checkmark$	$\checkmark$	$\checkmark$
JNLPBA [19]	$\checkmark$	$\times$	$\times$	$\checkmark$	$\checkmark$	$\checkmark$
LINNAEUS [20]	$\checkmark$	$\times$	$\times$	$\times$	$\times$	$\checkmark$
NCBI (disease) [21]	$\checkmark$	$\checkmark$	$\times$	$\checkmark$	$\checkmark$	$\checkmark$
Species-800 (S800) [22]	$\checkmark$	$\times$	$\times$	$\times$	$\times$	$\checkmark$
BC2GM [23]	$\checkmark$	$\times$	$\times$	$\checkmark$	$\checkmark$	$\checkmark$
DDI [24]	$\times$	$\times$	$\checkmark$	$\checkmark$	$\checkmark$	$\checkmark$
ChemProt [7]	$\checkmark$	$\checkmark$	$\checkmark$	$\checkmark$	$\checkmark$	$\checkmark$
i2b2 [25]	$\times$	$\times$	$\checkmark$	$\times$	$\times$	$\checkmark$
Euadr [26]	$\checkmark$	$\times$	$\times$	$\times$	$\times$	$\checkmark$
GAD [27]	$\checkmark$	$\times$	$\times$	$\checkmark$	$\checkmark$	$\checkmark$
BIOSSES [28]	$\times$	$\times$	$\checkmark$	$\checkmark$	$\times$	$\checkmark$
MedSTS [29]	$\times$	$\times$	$\checkmark$	$\times$	$\times$	$\checkmark$
MedNLI [30]	$\times$	$\times$	$\checkmark$	$\times$	$\times$	$\checkmark$
HoC [31]	$\times$	$\times$	$\checkmark$	$\checkmark$	$\times$	$\checkmark$
BioASQ 4b [32]	$\checkmark$	$\times$	$\times$	$\checkmark$	$\times$	$\checkmark$
BioASQ 5b [32]	$\checkmark$	$\times$	$\times$	$\checkmark$	$\times$	$\checkmark$
BioASQ 6b [32]	$\checkmark$	$\times$	$\times$	$\checkmark$	$\times$	$\checkmark$

Previous pre-trained LMs, including the work of Peng et al. [12], have common limitations: (1) these LMs are trained on limited domain-specific corpora (Table 1), whereas some tasks contain both clinical and biomedical terms, and therefore training with broader coverage of domain-specific corpora can improve performance; (2) by adopting BERT architecture, its’ training is slow and requires huge computational resources; and (3) all these LMs were demonstrated with selected BioNLP tasks (Table 2), and hence their generalizability is unproven.

In this study, we address the defined gaps in prior studies and hypothesize that training ALBERT that has been shown to be a superior model compared to BERT in NLP tasks [6] on both biomedical (PubMed and PMC) and clinical notes (MIMIC-III) corpora can be more effective and computationally efficient in a wide range of BioNLP tasks.

We present biomedical ALBERT (BioALBERT), a new LM designed and optimised to achieve benchmark performance on various BioNLP tasks. BioALBERT is based on the architecture of an ALBERT LM and is trained on a corpus of biomedical and clinical texts. We fine-tuned and compared the effectiveness of BioALBERT on 6 BioNLP tasks with 20 biomedical and clinical benchmark datasets with different sizes and complexity. Compared with most existing BioNLP LMs that are mainly focused on limited tasks, a large variant of BioALBERT trained on PubMed data achieved SOTA performance (BLURB score) on 5 out of 6 BioNLP tasks. Depending on the task, 5 different variants of BioALBERT outperformed previous SOTA models in 17 out of 20 tested datasets. BioALBERT achieved higher performance in NER, RE, Sentence similarity, Document classification and a higher Accuracy (lenient) score in QA than the current SOTA LMs. To facilitate developments in the important BioNLP community, we make the weights of pre-trained BioALBERT LMs publicly available.^1^

## Methods

BioALBERT has the same architecture as ALBERT and addresses the shortcomings of BERT-based biomedical models. First, BioALBERT uses cross-layer parameter sharing and reduces 110 million parameters of the 12-layer BERT-base model to 31 million parameters while keeping the same number of layers and hidden units. This is achieved by learning parameters for the first block and reusing the block in the remaining 11 layers. Secondly, BioALBERT uses sentence order prediction (SOP) loss that is designed to address the ineffectiveness of the next sentence prediction (NSP) loss used in the BERT. SOP enables the model to learn about discourse-level coherence characteristics from a finer-grained distinction and thus leads to better learning representation in downstream tasks. Thirdly, BioALBERT uses factorized embedding parameterization that decomposes the large vocabulary embedding matrix into two small matrices. This allows us to reduce the number of parameters between vocabulary and the first hidden layer. In BERT-based biomedical models, embedding size equals the hidden layer’s size. Lastly, BioALBERT is trained on massive biomedical corpora to be effective on BioNLP tasks to overcome the issue of the shift of word distribution from general domain corpora to biomedical corpora.

**Fig. 1:**
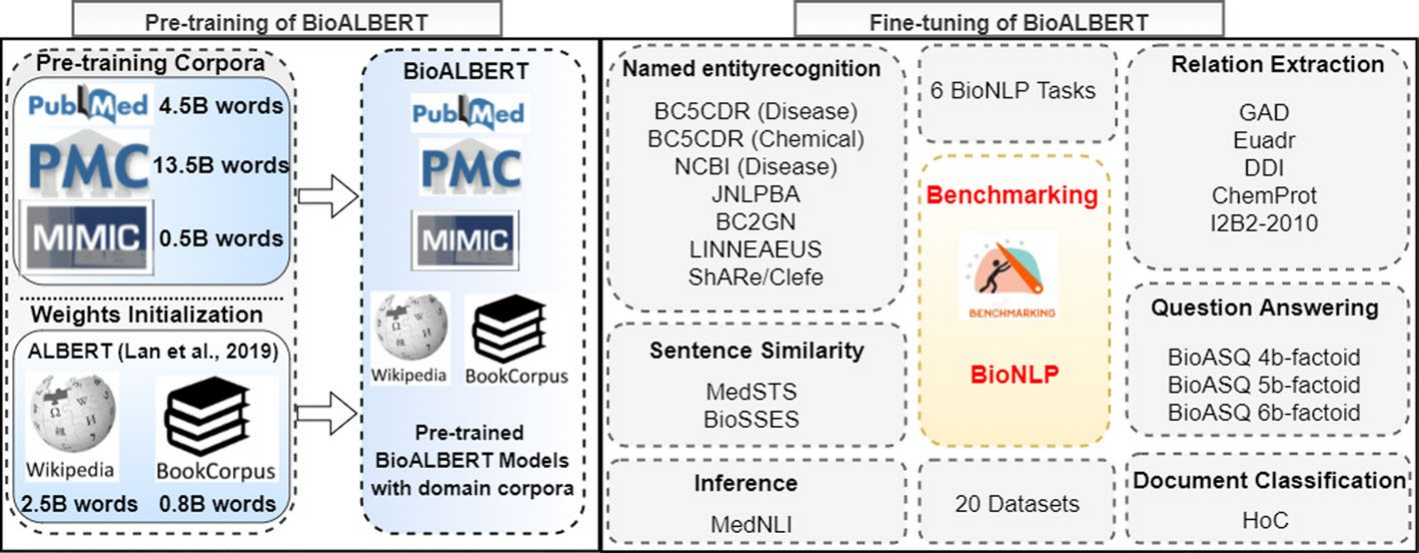
An overview of pre-training, fine-tuning and the diverse tasks and datasets present in Benchmarking for BioNLP using BioALBERT

Figure 1 depicts an overview of pre-training, fine-tuning, task variants, and datasets used in benchmarking BioNLP. We describe ALBERT and then the pre-training and fine-tuning process employed in BioALBERT.

### ALBERT

ALBERT [6] is built on the architecture of BERT to mitigate a large number of parameters in BERT, which causes model degradation, memory issues, and degraded pre-training time. ALBERT is a contextualised LM that is pre-trained using bidirectional transformers like BERT and is based on a masked language model (MLM). ALBERT employs an MLM to predict randomly masked words in a sequence and is capable of learning bidirectional representations.

ALBERT is trained on the same English Wikipedia and BooksCorpus as in BERT; however, it reduced BERT parameters by 87% and could be trained nearly twice as fast. ALBERT reduced parameter requirements by factorizing and decomposing a large vocabulary embedding matrix into two smaller matrixes. Other ALBERT enhancements include the use of SOP loss rather than NSP loss and the implementation of cross-layer parameter sharing, which keeps parameters from rising with the depth of the network. In the following section, we describe the steps involved in training BioALBERT.

### Pre-training BioALBERT

We first initialized BioALBERT with weights from ALBERT during the training phase. Biomedical terminologies have terms that could mean different things depending upon its context of appearance. For example, ER could be referred to ‘estrogen receptor’ gene or its product as protein. Similarly, RA may represent ‘right atrium’ or ‘rheumatoid arthritis’ depending upon the context of appearance. On the other hand, two terminologies could be used to refer to a similar concept, such as ‘heart attack’ or ‘myocardial infarction’. As a result, pre-trained LM trained on general corpus often obtains poor results.

BioALBERT is the first domain-specific LM trained on biomedical domain corpus and clinical notes. BioALBERT is trained on abstracts from PubMed, full-text articles of PMC, and clinical notes (MIMIC) and their combination. These unstructured and raw corpus were transformed to structured format by processing raw text files into a single sentence in which: (1) all blank lines within a text were deleted, and (2) any line with a length of fewer than 20 characters was removed. Overall, PubMed had 4.5 billion words, PMC had 13.5 billion, and MIMIC had 0.5 billion.

We used sentence embeddings for tokenization of BioALBERT by pre-processing the data as a sentence text. Each line was considered as a sentence keeping the maximum length to 512 words by trimming. If the sentence was shorter than 512 words, then more words were embedded from the next line. An empty line was used to define a new document. All of our models are trained with 3125 warm-up steps. We employed the LAMB optimizer to train our models and restricted the vocabulary size to 30K. During the training process, GeLU activation is employed in all variations of models. The training batch size for BioALBERT base models was 1024; however, due to computational resource constraints, the training batch size for BioALBERT large models was reduced to 256. Table 3 summarises the parameters used during the training stage.

**Table 3:** Summary of parameters used in the pre-training of BioALBERT

Summary of all parameters used: (pre-training)	
Architecture	ALBERT
Activation function	GeLU
Attention heads	12
No. of layers	12
Size of hidden layer	768
Size of embedding	128
Size of vocabulary	30k
Optimizer used	LAMB
Training batch size	1024 for base models 256 for large models
Evaluation batch size	16
Maximum sentence length	512
Maximum predictions per sentence	20
Warm-up steps	3125

Table 3 summarises the parameters used during the training stage.

We present 8 models (Table 4) consisting of 4 base and 4 large LMs. We observed that with a larger batch size during training, both base and large LMs were successful on the V3-8 TPU. The base model contained an embedding dimension of 128 and 12 million parameters, whereas the large model had an embedding dimension of 256 and 16 million parameters.

**Table 4:** BioALBERT trained on different training steps, different combinations of the text corpora, and BioALBERT model version and size

Model version	BioALBERT size	Combination of corpus used for training	Number of training steps
1	Base1	Wikipedia + BooksCorpus + PubMed	200K
2	Base2	Wikipedia + BooksCorpus + PubMed+ PMC	470K
3	Large1	Wikipedia + BooksCorpus + PubMed	200K
4	Large2	Wikipedia + BooksCorpusPubMed + PMC	470K
5	Base3	Wikipedia + BooksCorpus + PubMed + MIMIC-III	200K
6	Base4	Wikipedia + BooksCorpus + PubMed + PMC + MIMIC-III	200K
7	Large3	Wikipedia + BooksCorpus + PubMed + MIMIC-III	270K
8	Large4	Wikipedia + BooksCorpus + PubMed + PMC + MIMIC-III	270K

### Fine-tuning BioALBERT

Similar to other SOTA biomedical LMs,^2^ BioALBERT was tested on a number of downstream BioNLP tasks which required minimal architecture alteration. BioALBERT’s computational requirements were not significantly large compared to other baseline models, and fine-tuning only required relatively small computation compared to the pre-training. BioALBERT employed reduced physical memory, improved parameter sharing approaches, and learned word embeddings via sentence piece tokenization, giving it higher performance and faster training than existing SOTA biomedical LMs.

We used the weights of the pre-trained BioALBERT LM during fine-tuning. We used an AdamW optimizer with a learning rate of 0.00001. During training, a batch size of 32 was used. In the NER task, we fixed the length of sentences to 512, whereas, for the remaining 5 tasks, we used a sentence length of 128 in our experiments. Further, we lower-cased all words. Finally, we fine-tuned BioALBERT using 10k training steps and 320 warm-up steps. The test splits were used for prediction, and the evaluation metric was compared with previous SOTA models. Table 5 summarises all fine-tuning parameters.

**Table 5:** Summary of parameters used in fine-tuning

Summary of all parameters used: (fine-tuning)	
Optimizer used	AdamW
Training batch size	32
Checkpoint saved	500
Learning rate	0.00001
Training steps	10k
Warm-up steps	320

### Experimental settings

We tested with different experimental settings during the pre-training and fine-tuning stages. Our experiments produced best results using the parameters summarised Table 3 for pre-training, and Table 5 for fine-tuning.

### Tasks and datasets

We fine-tuned BioALBERT on 6 different BioNLP tasks with 20 datasets that cover a wide variety of data quantities and challenges (Table 6). We rely on pre-existing datasets that are widely supported in the BioNLP community and describe each of these tasks and datasets.

**Table 6:** Statistics of the datasets used

Dataset	Task	Domain	Train	Dev	Test	Metric
BC5CDR (disease)	NER	Biomedical	109,853	121,971	129,472	F1-Score
BC5CDR (chemical)	NER	Biomedical	109,853	117,391	124,676	F1-Score
NCBI (disease)	NER	Clinical	135,615	23,959	24,488	F1-Score
JNLPBA	NER	Biomedical	443,653	117,213	114,709	F1-Score
BC2GM	NER	Biomedical	333,920	70,937	118,189	F1-Score
LINNAEUS	NER	Biomedical	267,500	87,991	134,622	F1-Score
Species-800 (S800)	NER	Biomedical	147,269	22,217	42,287	F1-Score
Share/Clefe	NER	Clinical	4628	1075	5195	F1-Score
GAD	RE	Biomedical	3277	1025	820	F1-Score
Euadr	RE	Biomedical	227	71	57	F1-Score
DDI	RE	Biomedical	2937	1004	979	F1-Score
ChemProt	RE	Biomedical	4154	2416	3458	F1-Score
i2b2	RE	Clinical	3110	11	6293	F1-Score
HoC	Document classification	Biomedical	1108	157	315	F1-Score
MedNLI	Inference	Clinical	11,232	1395	1422	Accuracy
MedSTS	Sentence similarity	Clinical	675	75	318	Pearson
BIOSSES	Sentence similarity	Biomedical	64	16	20	Pearson
BioASQ 4b-factoid	QA	Biomedical	327	–	161	Accuracy (Lenient)
BioASQ 5b-factoid	QA	Biomedical	486	–	150	Accuracy (Lenient)
BioASQ 6b-factoid	QA	Biomedical	618	–	161	Accuracy (Lenient)

- *Named entity recognition (NER)* Recognition of proper domain-specific nouns in a biomedical corpus is the most basic and important BioNLP task. The F1-score was adopted as a NER evaluation metric. BioALBERT was evaluated on 8 NER benchmark datasets (From Biomedical and Clinical domain): We used NCBI (Disease) [21], BC5CDR (Disease) [18], BC5CDR (Chemical) [18], BC2GM [23], JNLPBA [19], LINNAEUS [20], Species-800 (S800) [22] and Share/Clefe [17] datasets.
- *Relation extraction (RE)* RE tasks aim to identify relationship among entities in a sentence. The annotated data were compared with relationship and types of entities. As an evaluation metric, the micro-average F1-score metric was used. For RE, we used DDI [24], Euadr [26], GAD [27], ChemProt [7] and i2b2 [25] datasets.
- *Document classification* Document classification tasks classify the whole document into various categories. Multiple labels from texts are predicted in the multi-label classification task. We followed standard practice and reported the F1-score for the document classification task. For document classification, we used HoC (the hallmarks of Cancers) [31] dataset.
- *Inference* Inference tasks determine if the premise sentence implies the hypothesis sentence. It mainly focuses on causation relationships between sentences. For evaluation, we used overall standard accuracy as a metric. For inference, we used MedNLI [30] dataset.
- *Sentence similarity (STS)* STS task is to predict similarity scores by estimating whether two sentences deliver similar contents. We used Pearson correlation coefficients to assess similarity, as is standard. We used MedSTS [29] and BIOSSES [28] datasets for sentence similarity task.
- *Question answering (QA)* QA is the task of answering questions asked in the natural language given relevant passages. We used accuracy as an evaluation metric for the QA task. For QA, we used BioASQ factiod [32] datasets.

## Results and discussion

- *Comparison with SOTA biomedical LMs* Table 7 summarises the results^3^,^4^ We observe that the performance of BioALBERT^5^ is higher than SOTA models in 5 out of the 6 tasks. Overall, a large version of BioALBERT that is trained on PubMed abstract achieved the best results among all the tasks. To be precise, depending on tasks, 5 different variants of BioALBERT outperformed previous SOTA models in 17 out of 20 tested datasets.

For NER, BioALBERT was significantly higher compared to SOTA methods on all 8 datasets (ranging from 4.61 to 23.71%) and outperformed the SOTA models by 11.09% in terms of micro averaged F1-score (BLURB score). For, Share/Clefe dataset, BioALBERT increased the performance by 19.44%, 10.63% for BC5CDR-disease, 4.61% for BC5CDR-chemical, 4.74% for JNLPBA, 6.19% for Linnaeus, 7.47% for NCBI-disease, 23.71% and 12.25% for S800 and BC2GM datasets, respectively.

**Table 7:** Comparison of BioALBERT versus SOTA methods in BioNLP tasks

Dataset	SOTA	BioALBERT								Difference over SOTA
		Base1	Base2	Large1	Large2	Base3	Base4	Large3	Large4	
*Named entity recognition task*										
Share/Clefe	75.40	94.27	94.47	93.16	94.30	94.84*	94.82	94.70	94.66	19.44 $\uparrow$
BC5CDR (disease)	87.15	97.66	97.62	97.78*	97.61	90.03	90.01	90.29	91.44	10.63 $\uparrow$
BC5CDR (chemical)	93.47	97.90	98.08*	97.76	97.79	89.83	90.08	90.01	91.48	4.61 $\uparrow$
JNLPBA	82.00	82.72	83.22	84.01	83.53	86.74*	86.56	86.20	85.72	4.74 $\uparrow$
Linnaeus	93.54	99.71	99.72	99.73	99.73*	95.72	98.27	98.24	98.23	6.19 $\uparrow$
NCBI (disease)	89.71	95.89	95.61	97.18*	95.85	85.82	85.93	85.86	85.83	7.47 $\uparrow$
S800	75.31	98.76	98.49	99.02*	98.72	93.53	93.63	93.63	93.63	23.71 $\uparrow$
BC2GM	85.10	96.34	96.02	96.97*	96.33	83.35	83.38	83.44	84.72	11.87 $\uparrow$
BLURB	84.61	95.41	95.41	95.70*	95.48	89.98	90.34	90.30	90.71	11.09$\uparrow$
*Relation extraction task*										
DDI	82.36	82.32	79.98	83.76	84.05*	76.22	75.57	76.28	76.46	1.69 $\uparrow$
ChemProt	77.50	78.32*	76.42	77.77	77.97	62.85	62.34	61.69	57.46	0.82 $\uparrow$
i2b2	76.40	76.49	76.54	76.86*	76.81	73.83	73.08	72.19	75.09	0.46 $\uparrow$
Euadr	**86.51**	82.32	74.07	84.56	81.32	62.52	76.93	70.41	70.48	− 1.95 $\downarrow$
GAD	**84.30**	73.82	66.32	76.74	69.65	72.68	69.14	71.81	68.17	− 7.56 $\downarrow$
BLURB	79.14	78.66	74.67	79.94*	77.96	69.62	71.41	70.50	69.53	0.80$\uparrow$
*Sentence similarity task*										
BIOSSES	92.30	82.27	73.14	92.80*	81.90	24.94	55.80	47.86	30.48	0.50 $\uparrow$
MedSTS	84.80	85.70	85.00	85.70*	85.40	51.80	56.70	45.80	42.00	0.90 $\uparrow$
BLURB	88.20	83.99	79.07	89.25*	83.65	38.37	56.25	46.83	36.24	1.05$\uparrow$
*Inference task*										
MedNLI	**84.00**	77.69	76.35	79.38	79.52	78.25	77.20	76.34	75.51	− 4.48 $\downarrow$
*Document classification task*										
HoC	87.30	83.21	84.52	87.92*	84.32	64.20	75.20	61.00	81.70	0.62 $\uparrow$
*Question answering task*										
BioASQ 4b	47.82	47.90	48.34	48.90*	48.25	47.10	47.35	45.90	46.10	1.08 $\uparrow$
BioASQ 5b	60.00	61.10	61.90	62.31*	61.57	58.54	59.21	58.98	58.50	2.31 $\uparrow$
BioASQ 6b	57.77	59.80	62.00	62.88*	61.54	56.10	56.22	56.60	56.85	5.11 $\uparrow$
BLURB	55.20	56.27	57.41	58.03*	57.12	53.91	54.26	53.83	53.82	2.83$\uparrow$

The ‘difference over SOTA’ indicate the absolute change ($\uparrow$ for increase and $\downarrow$ for decrease) in metric performance over SOTA. Bold is the best results. We present the SOTA model performances on several datasets as follows: (1) JNLPBA, BC2GM, ChemProt, and GAD from Yuan et al. [15] (KeBioLM), (2) DDI and BIOSSES are from Gu et al. [14] (PubMedBERT), (3) Share/Clefe, i2b2, MedSTS, MedNLI and HoC from Peng et al. [12] (BLUE), (4) BC5CDR (disease), BC5CDR (chemical), NCBI (Disease), S800, Euadr, BioASQ 4b, BioASQ 5b, and BioASQ 6b, from Lee et al. [13] (BioBERT), and (5) LINNAEUS from Giorgi and Bader [33]. The biomedical language understanding and reasoning benchmark (BLURB) is an average score among all tasks used in previous studies [14, 15]

*Indicates that BioALBERT (bold) achieved a significant $(\textit{p} < 0.05)$ performance improvement over SOTA model under one-sample t-test

For RE, BioALBERT outperformed SOTA methods on 3 out of 5 datasets by 1.69%, 0.82%, and 0.46% on DDI, ChemProt and i2b2 datasets, respectively. On average (micro), BioALBERT obtained a higher F1-score (BLURB score) of 0.80% than the SOTA LMs. For Euadr and GAD performance of BioALBERT slightly drops because the splits of data used are different. We used an official split of the data provided by authors, whereas the SOTA method reported the results using 10-fold cross-validation.

For STS, BioALBERT achieved higher performance on both datasets by a 1.05% increase in average Pearson score (BLURB score) as compared to SOTA models. In particular, BioALBERT achieved improvements of 0.50% for BIOSSES and 0.90% for MedSTS.

Similarly, for document classification, BioALBERT slightly increase the performance by 0.62% for the HoC dataset and the inference task (MedNLI dataset), the performance of BioALBERT drops slightly, and we attribute this to the average length of the sentence being smaller compared to others.

For QA, BioALBERT achieved higher performance on all 3 datasets and increased average accuracy (lenient) score (BLURB score) by 2.83% compared to SOTA models. In particular, BioALBERT improves the performance by 1.08% for BioASQ 4b, 2.31% for BioASQ 5b and 5.11% for BioASQ 6b QA datasets respectively as compared to SOTA.

Thus, we conclude that our results validate our hypothesis that training ALBERT that addresses limitations of BERT on biomedical and clinical notes is more effective and computationally faster compared to other biomedical language models.

We note that the performance of ALBERT (both base and large), when pre-trained on MIMIC-III, in addition to PubMed and combination of PubMed and PMC, drops as compared to the same pre-trained ALBERT without MIMIC-III, especially in RE, STS, and QA tasks. We attribute this to the following observations (1) clinical (MIMIC-III) data consists of notes from the ICU of Beth Israel Deaconess Medical Center (BIDMC) only, the data size is small (0.5 billion words) compared to the biomedical (PubMed + PMC) data (18 billion words); and (2) problem of bias in a training data. For instance, in MIMIC-III, heart disease is more common in males compared to females—an example of gender bias is that there are fewer clinical studies involving black patients compared to other groups—an example of ethnicity bias. Based on these observations, we suggest that in future works it is necessary to identify and reduce any form of bias that allows the model to make fair decisions without favoring any group. Further, clinical notes differ substantially from biomedical literature. Consequently, models pretrained on clinical notes perform poorly on biomedical tasks; therefore, it is advantageous to create separate benchmarks for these two domains.

## Analysis

- *Run-time statistics* We compared pre-training run-time statistics of BioALBERT with BioBERT. We demonstrated that all the variants of BioALBERT outperformed BioBERT. The difference in performance is significant, identifying BioALBERT as a robust and practical model. ${\text {BioBERT}}_{{Base1}}$ trained on PubMed took 10 days, and ${\text {BioBERT}}_{{Base2}}$ trained on PubMed and PMC took 23 days, whereas all models of BioALBERT took less than 5 days for training an equal number of steps. Table 8 shows the run-time statistics for both pre-trained LMs.

**Table 8:** Comparison of run-time (in days) statistics of BioALBERT versus BioBERT

Model	Training time (in days)
${\text {BioBERT}}_{{Base2}}$	23.00
${\text {BioBERT}}_{{Base1}}$	10.00
${\text {BioALBERT}}_{{Base1}}$	3.00
${\text {BioALBERT}}_{{Base2}}$	4.08
${\text {BioALBERT}}_{{Large1}}$	2.83
${\text {BioALBERT}}_{{Large2}}$	3.88
${\text {BioALBERT}}_{{Base3}}$	4.02
${\text {BioALBERT}}_{{Base4}}$	4.45
${\text {BioALBERT}}_{{Large3}}$	4.62
${\text {BioALBERT}}_{{Large4}}$	4.67

Refer to Table 4 for more details of BioALBERT size. ${\text {BioBERT}}_{{Base1}}$ and ${\text {BioBERT}}_{{Base2}}$ refers to BioBERT trained on PubMed and PubMed+PMC respectively

**Fig. 2:**
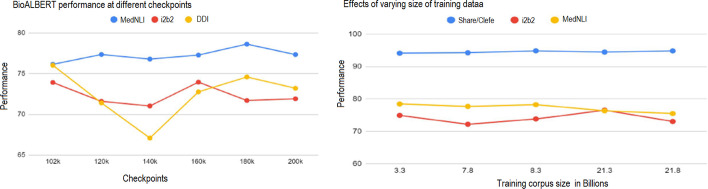
Performance of BioALBERT at different checkpoints (left) and effects of varying the size of the PubMed corpus for pre-training (right)

**Fig. 3:**
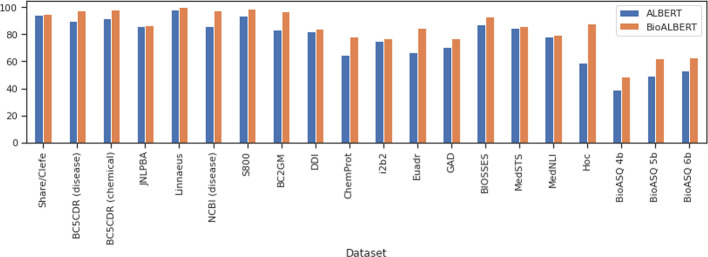
Comparison of BioALBERT versus ALBERT. The evaluation scale is same as previously reported in Table 7

- *Effect of using additional training data* We used additional corpora of different sizes for training and investigated their effect on performance. For the BioALBERT base model trained on the combination of PubMed, PMC, and MIMIC-III, we set the number of pre-training steps to 200K and varied the training corpus size. We saved the pre-trained weights from BioALBERT at different pre-training steps to measure how the number of pre-training steps affects its performance on fine-tuning tasks. Figure 2 (left) shows the performance changes on the same three datasets with the number of pre-training steps. Further, Fig. 2 (right) shows that the performance on three datasets (share/clefe, i2b2, MedNLI) reaches optimal performance when trained on 3 billion words and performance slightly varies when we increase the size of the training corpus. These results demonstrate that choosing the right size of training data and pre-trained checkpoints are important to achieve the optimal performance for BioNLP tasks.
- *BioALBERT versus ALBERT* We compared the performance of ALBERT trained on general corpora to BioALBERT with the results shown in Fig. 3. We fine-tuned ALBERT on downstream tasks the same way we fine-tuned BioALBERT. BioALBERT consistently achieved higher performance on all 6 tasks (20 out of 20 datasets) compared to ALBERT. Additionally, as shown in Table 9, we evaluated ALBERT and BioALBERT predictions to determine the effect of pre-training on NER and HoC tasks. For NER, we observed that although the gains of BioALBERT are small compared to ALBERT, BioALBERT can better recognise the biomedical entities compared to ALBERT in both JNLPBA and Share/Clefe datasets. Similarly, for HoC data, BioALBERT can better recognise biomedical entities compared to ALBERT. We attribute the increase in performance of BioALBERT to a word distribution shift from general domain corpora to biomedical corpora in the BioNLP task. The analysis presented in Fig. 3 and Table 9 validates our hypothesis that training ALBERT on biomedical corpora improves the performance compared to LMs trained on LM.

**Table 9:** Prediction samples from ALBERT and BioALBERT

Dataset	Model	Sample
JNLPBA	ALBERT	Number of glucocoticoid receptors in lymphocytes and their sensitivity to...
	BioALBERT	Number of glucocoticoid **receptors** in **lymphocytes** and their sensitivity to...
Share/Clefe	ALBERT	The mitral valve leaflets are mildly thickened. There is mild mitral annular calcification. TRICUSPID VALVE...
	BioALBERT	The mitral valve leaflets are mildly **thickened**. There is mild **mitral annular calcification**. TRICUSPID VALVE...
HoC	ALBERT	In contrast, 15 Gy increased the expression of p27 in radiosensitive tumors and reduced it in radioresistant tumors
	BioALBERT	In contrast, 15 Gy increased the expression of p27 in radiosensitive **tumors** and reduced it in **radioresistant tumors**

Bold entities are better recognised by BioALBERT

## Limitations and future directions

Although domain-specific LMs have improved the performance for BioNLP tasks, there are several limitations that warrant future work. In supervised machine learning, pre-training of domain-specific LMs requires a large volume of domain-specific corpora and expensive computational resources such as GPUs/TPUs for longer pre-training duration [34]. To address these challenges, there is a need for time-efficient and low-cost methods. One of these methods is self-supervised learning (SSL) [35] which learns from unlabeled data. SSL could be one of the future directions to explore to overcome these limitations using transfer learning. Another emerging area is exploring generalized zero-shot learning (GZSL) [36] where the training classes are presented only at test time. Further, the performance of domain-specific LMs can be improved by reducing biases and injecting human-curated knowledge bases [37].

## Conclusion

We present BioALBERT, the first adaptation of ALBERT trained on both biomedical text and clinical data. Our experiments show that training general domain language models on domain-specific corpora result in an increase in performance across a range of biomedical BioNLP tasks. A large variant of BioALBERT trained on PubMed outperforms previous state-of-the-art models on 5 out of 6 benchmark BioNLP tasks. We expect that the release of the BioALBERT models and data will support the development of new applications built from biomedical NLP tasks.

## Data Availability

Pre-trained weights of BioALBERT models together with the datasets analysed in this paper are available at https://github.com/usmaann/BioALBERT. The PubMed data are available at https://www.ncbi.nlm.nih.gov/pubmed/. The PMC data are available at https://www.ncbi.nlm.nih.gov/pmc/. The MIMIC data are available at https://mimic.mit.edu/.

## Footnotes

1 https://github.com/usmaann/BioALBERT.

2 We followed the same architectural modification as previous studies in the downstream task.

3 Refer to Table 4 for more details of BioALBERT size and training corpus and Table 6 for the evaluation metric used in each dataset. for all the BioALBERT variants in comparison to the baselines when fine-tuned on tested datasets.

4 The baseline results were obtained from the original study.

5 Here, we discuss the best model of BioALBERT Out of 8 versions of BioALBERT.
